# Biowep: a workflow enactment portal for bioinformatics applications

**DOI:** 10.1186/1471-2105-8-S1-S19

**Published:** 2007-03-08

**Authors:** Paolo Romano, Ezio Bartocci, Guglielmo Bertolini, Flavio De Paoli, Domenico Marra, Giancarlo Mauri, Emanuela Merelli, Luciano Milanesi

**Affiliations:** 1 Bioinformatics and Structural Proteomics, National Cancer Research Institute (IST), Genova, Italy; 2 Department of Mathematics and Computer Science (DMI), University of Camerino, Camerino (MC), Italy; 3 Department of Informatics, Systems and Communication (DISCo), University of Milan "Bicocca", Milano, Italy; 4 Biomedical Technologies Institute (ITB), National Research Council, Milano, Italy; 5 CILEA, Segrate (MI), Italy

## Abstract

**Background:**

The huge amount of biological information, its distribution over the Internet and the heterogeneity of available software tools makes the adoption of new data integration and analysis network tools a necessity in bioinformatics. ICT standards and tools, like Web Services and Workflow Management Systems (WMS), can support the creation and deployment of such systems. Many Web Services are already available and some WMS have been proposed. They assume that researchers know which bioinformatics resources can be reached through a programmatic interface and that they are skilled in programming and building workflows. Therefore, they are not viable to the majority of unskilled researchers. A portal enabling these to take profit from new technologies is still missing.

**Results:**

We designed biowep, a web based client application that allows for the selection and execution of a set of predefined workflows. The system is available on-line. Biowep architecture includes a Workflow Manager, a User Interface and a Workflow Executor. The task of the Workflow Manager is the creation and annotation of workflows. These can be created by using either the Taverna Workbench or BioWMS. Enactment of workflows is carried out by FreeFluo for Taverna workflows and by BioAgent/Hermes, a mobile agent-based middleware, for BioWMS ones. Main workflows' processing steps are annotated on the basis of their input and output, elaboration type and application domain by using a classification of bioinformatics data and tasks. The interface supports users authentication and profiling. Workflows can be selected on the basis of users' profiles and can be searched through their annotations. Results can be saved.

**Conclusion:**

We developed a web system that support the selection and execution of predefined workflows, thus simplifying access for all researchers. The implementation of Web Services allowing specialized software to interact with an exhaustive set of biomedical databases and analysis software and the creation of effective workflows can significantly improve automation of in-silico analysis. Biowep is available for interested researchers as a reference portal. They are invited to submit their workflows to the workflow repository. Biowep is further being developed in the sphere of the Laboratory of Interdisciplinary Technologies in Bioinformatics – LITBIO.

## Background

Integration of data and processes needs stability of the knowledge domain. This implies "a-priori" deep knowledge of the domain and well defined information and data, both leading to a standardization of information schemas and formats. Also, essential is a clear definition of the goals of integration processes. On the contrary, integration fears heterogeneous data and systems, uncertain domain knowledge, highly specialized and quickly evolving information, lacking of predefined, clear goals and originality of procedures and processes. In biology, a pre-analysis and reorganization of the data is very difficult, because data and related knowledge change very quickly. Moreover, complexity of information makes it difficult to design data models which can be valid for different domains and over time. Finally, goals and needs of researchers evolve very quickly according to new theories and discoveries, this leads to frequent new procedures and processes.

Current integration methods, that are based on syntactical tools like explicit cross-references, implicit links (e.g., through names of biological entities) and common contents (achieved by using common vocabularies, reference lists and lexicons) are inadequate. Instead, new methods based on semantic links, such as those that can be derived by using metadata descriptions and reference ontologies, seem more adequate. Flexibility of systems, including the ability to support frequent changes of data, software and analysis, is mandatory.

Integration of heterogeneous data is anyway needed to achieve a better and wider view of all available information, to automatically carry out analysis and/or searches involving more databases and software and to perform analysis involving large data sets. Finally, only a tight integration of data and analysis tools can lead to a real data mining. In such a context, the need is felt for a system that is able to improve the information accessibility.

Among current ICT technologies, workflow management systems, in connection with Web Services, seem to be the most promising ones. Reasons for the setting up of Web Services in bioinformatics have already been presented [1,2]. These include the need for overcoming the scaling problem arising from the use of high-throughput experimental protocols that provide such huge results that their analysis needs a "high-throughput" process in order to be studied in an adequate time scale. This could not be achieved through the traditional approach implying manual access to web sites. Instead, software driven access to Web Services implementations of the required analysis software could achieve it. Also, WS would offer bioinformatics the possibility of implementing a really distributed analysis environment, while protecting intellectual property rights for data, algorithms and source code, that would not be copied and would remain on the owners' information system.

WS have already been implemented by many Institutes and service centres in the biomedical field. Partial lists of Web Services for bioinformatics are available at the myGrid Wiki site [3] and in the Taverna web site [4]. Also, Web Services can be retrieved and accessed through the MOBY Central, an archive based on BioMOBY, an open source software that implements an architecture for the discovery and distribution of biological data through Web Services [5].

Web Services alone are not sufficient for automating "in-silico" bioinformatics processes: the notion of workflow must also be included. Workflows are defined as "computerized facilitations or automations of a business process, in whole or part" (Workflow Management Coalition, WfMC) [6]. Their goal is the implementation of data analysis processes in standardized environments and their main advantages relate to effectiveness, reproducibility, reusability of intermediate results and traceability. Effectiveness is achieved through automation of repetitive procedures: being an automatic procedure, a workflow can free bio-scientists from repetitive interactions with the web, at the same time supporting good practice. Reproducibility is also granted by the implementation of repetitive procedures, although it is limited, in biology, by the frequent update of information sources; anyway, analyses can be replicated over time. Reusability is implemented by storing intermediate results and by allowing their use in subsequent workflows executions. Finally, traceability is achieved by storing intermediate results and allowing their analysis: the workflow is then carried out in a transparent analysis environment where data provenance can be checked and/or controlled. This is especially important when unexpected data are obtained.

Workflow management systems should not be compared to other integration systems, such as the Sequence Retrieval System (SRS, [7-9]) since they carry out tasks that are quite different. While SRS is able to perform limited, predefined operations (i.e., boolean and linking operations) on a local set of databases, a workflow management system is able to carry out any kind of elaborations and analysis on remote databases. Instead, an SRS site could be remotely queried through a properly programmed Web Service and its abilities, such as querying more databases at the same time, could therefore be added to a workflow. With workflow management systems, query processing on multiple sources can be achieved by carrying out parallel searches and later merging results. Alternate processing is also available with workflow management systems. This can be achieved by assigning the same task in a workflow to more services, by also providing them priority levels, and by invoking the services having the highest priority level first. Services with lower priority levels can then be invoked, if and when the previously called ones should fail.

Many workflow management systems have been proposed in the biomedical domain. Some of them are add-ons to other tools, some are autonomous applications. Among open source applications developed by academia, Taverna Workbench [10] from the European Bioinformatics Institute (EBI) is the most well known. Taverna has been developed in the frame of the myGrid project [11]. It is able to build complex analysis workflows, to access both remote and local processors of various kinds, to launch execution of workflows and to display different types of results, including text, web pages and various kinds of images. Workflows execution is carried out by an associated tool, the FreeFluo enactor engine. Processors that can be used through the Taverna Workbench include Web Services, either described through their WSDL definition or accessed through a bioMOBY registry, and retrieval of information from BioMart databases [12-14]. Local processors are also included with Taverna for basic elaborations such as simple list or string processing, definition of constant values, local input/output management. New local elaborations can be further defined and specialised by the user that is allowed to create and add scripts by using BeanShell (Lightweight Scripting for Java) [15].

All workflow management systems developed so far assume that end users know all bioinformatics resources they need, especially those resources that can be reached through a programmatic interface, and are proficient, if not skilled, in programming computers and in the composition of their own workflows. They are therefore not viable to the vast majority of biologists and researchers that are normally only skilled in the use of web interfaces.

A portal enabling the vast majority of unskilled researchers to take profit from these new technologies is still missing. A workflow enactment portal should provide end users with a user-friendly, personalized tool where he/she can register his/her personal preferences and interests, easily identify workflows and keep a record of the results. The ideal portal would also be able to enact workflows that are available through all workflow management systems. A first attempt was carried out within the Oncology over Internet (O_2_I) project [16] that led to the design of an architecture for a user interface. We present here biowep, a workflow enactment portal for bioinformatics, that is an actual implementation of an extension of design principles defined within the O_2_I project. Biowep manages profiles of users, allows for searching in the repository of workflows, supports selection and execution of predefined workflows and allows for storing interesting results. It presents a user-friendly web interface and it therefore is viable to all end users, by also allowing them to take profit from all advantages of the workflow management systems.

## Results and discussion

We designed biowep, a workflow enactment portal (web based client application, as defined in the WfMC Reference Model), that allows for the selection and execution of a set of predefined, annotated workflows. The system is available on-line [17].

Biowep has been implemented starting from the architecture designed in the Oncology over Internet – O_2_I project, but it is:

• not restricted to oncology, all bioinformatics applications can be included,

• not limited to workflows created by using the Taverna Workbench: two workflows management systems and enactment engines are presently used and more can be added,

• not limited to internally created workflows, since submission of workflows for insertion in biowep is allowed.

Of course, such generalization has implied changes in the initial architecture. Such changes have been taken into account during the implementation of the portal.

Furthermore, end users support has been implemented and the software has been made available to interested researchers under the GNU Less General Public License (LGPL) license.

### Selection of a workflow

In biowep, users are authenticated. The system stores information on workflows executed by each user and it is therefore able to list workflows executed by him/her. Last executed workflows are listed first and for each workflow executed in the past, its version number and the execution date are also shown.

**Figure 1:**
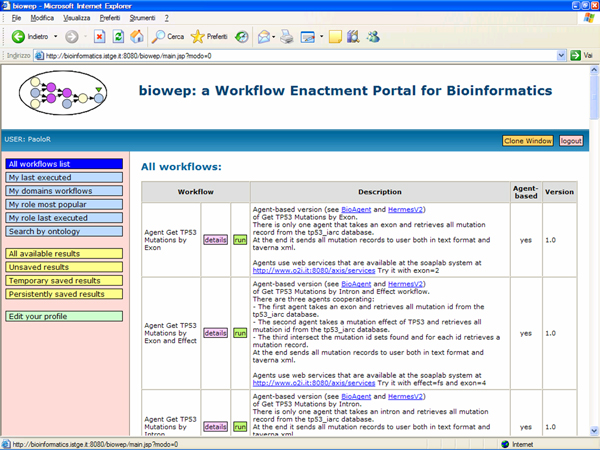
**List of workflows available in the biowep workflow repository**. In this figure, the web page listing all workflows is shown. Each item in the list includes the name of the workflow, a short description of the same, its type (this information also determines which engine must execute it) and its current version. Also available for each item are two buttons: one for launching the workflow and one for displaying its detailed description.

The system also supports retrieval of lists of available workflows on the basis of the role of the user in his/her organization (e.g., researcher, clinician, computer scientist) and of the domains of interest (e.g., mutation analysis, gene prediction). In the latter case, workflows are listed by date (last executed first), while, in the former, they can also be listed by number of executions carried out by all users (i.e., by popularity among users of the system). In figure 1, the web page listing all workflows available in the workflow repository is shown.

The list includes the name and a short description of the workflows, together with their current version number and the last execution date. In these pages, two buttons are always available for enacting the workflow (button 'run') or retrieving its details (button 'details').

**Figure 2:**
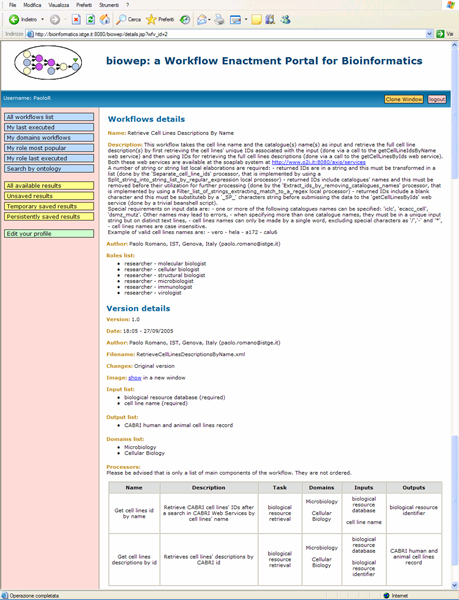
**Details on a workflow included in the repository**. In this figure, the web page including the detailed description of a workflow is shown. This description includes information both at the workflow and at the version levels. The first level has data such as the author and the run contexts, while the second level includes, among other information, the annotation of the workflow. A link to the diagram of the workflow is also available.

In figure 2, details of a workflow are shown. These include its overall annotation and the annotation of its main steps. Also available is a link to a diagram of the workflow.

### Search of a workflow through its annotation

**Figure 3:**
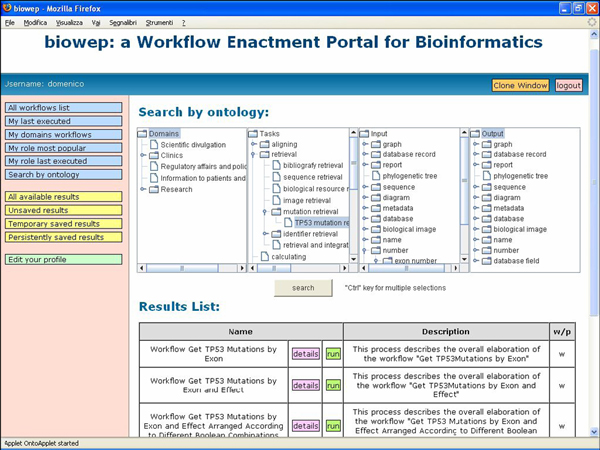
**"Search by ontology" page, allowing to search workflows by their annotations**. In this figure, the web page allowing for the "search by ontology" is shown. Columns include, from left to right, the application domain, the task, the input and the output. Multiple conditions that are set on each column are combined by OR, and conditions that are imposed on different columns are combined by AND. Results of the search are displayed in the lower part of the same page and include both workflows and single steps.

Search and identification of workflows of interest can also be achieved by means of the annotation of the workflows. In figure 3, the web page allowing this kind of search is shown. Conditions can be defined on the application domain of the workflow, as well as on its type (the kind of elaboration or analysis that it performs) and the type of its input and output fields. Conditions can be set on each column (see figure 3 again) and they are then combined by using a logical AND. When multiple conditions are put on the same column, these are combined by using a logical OR. An example query could be: find all workflows in the molecular biology domain (application domain) including at least one elaboration step that retrieves (retrieval task) DNA sequences (output) on the basis of a Genbank accession number (input). Of course, end users are not obliged to put conditions on every field: these can be left undetermined. A search that does not impose any condition on any field will result in a list of all annotated steps and workflows.

Results are listed in the same page and include the annotation. Also included is a note that specifies whether the retrieved data refers to the overall task performed by a workflow or to the task performed by a single step in a workflow. In the former case, the workflow can be enacted, while, in the latter, a list of all workflows including that step can be requested.

### Workflow enactment

**Figure 4:**
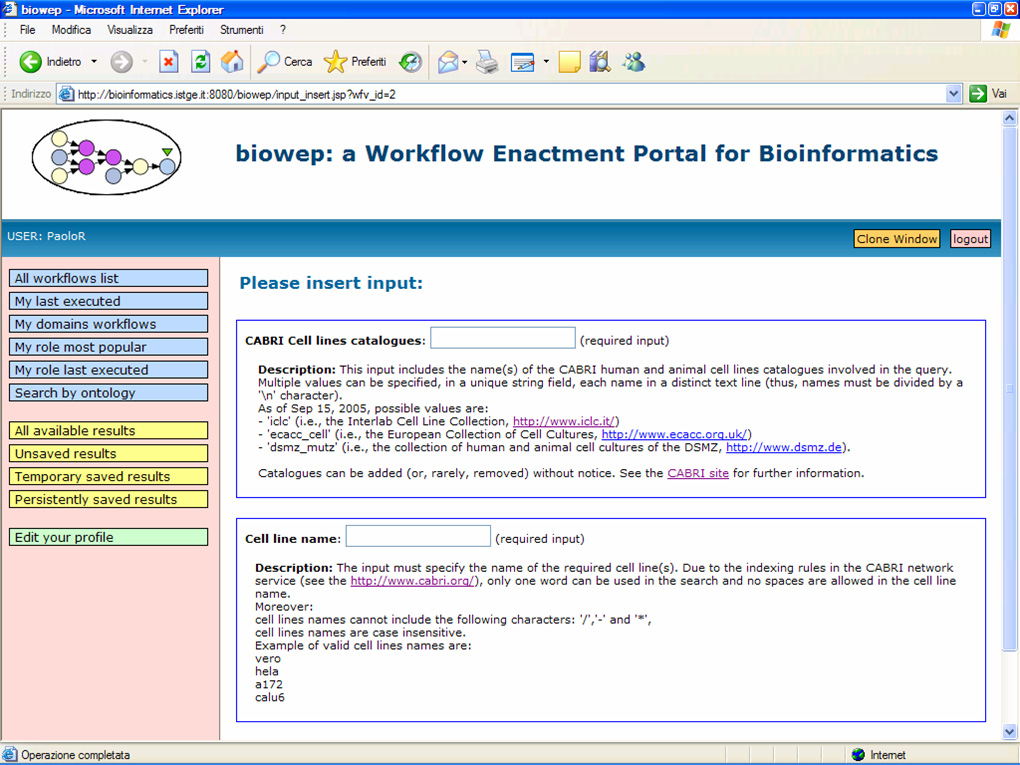
I**nput form for executing a workflow**. In this figure, the input form for the execution of a workflow is shown. Input fields are described in details and suggestions for input values are given. Required and optional fields are pointed out.

In figure 4, the input form for the execution of a workflow is shown. In this page, input fields are described in details and suggestions for possible input values are reported, so that the required data syntax is clearly shown. Required and optional fields are pointed out.

The enactment of workflows created with Taverna is carried out, as already said, by using Freefluo. In this case, the execution is performed on the server and results are stored in the system and made available to the user as soon as they are available. If the execution takes more time than a predefined period (usually 30 seconds, but this time can be changed by modifying a parameter in the configuration file), the workflow is executed in background and the user is invited to retrieve results later in the results section. In this case, results are also returned by email. Instead, workflows created with BioWMS, are executed by issuing a request to the Hermes server that is available at the University of Camerino. In this case, results are only returned by email.

### Visualization and management of results

**Figure 5:**
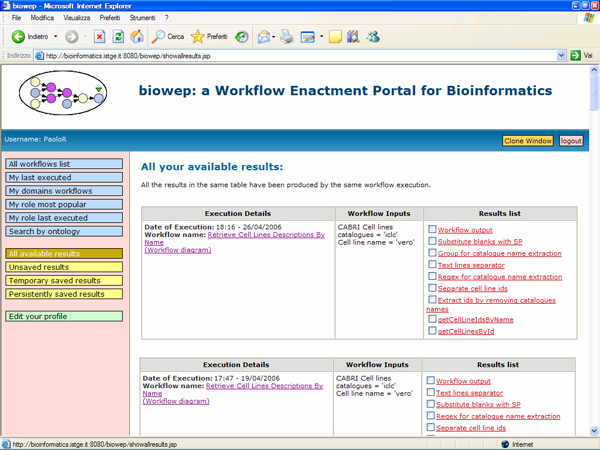
**List of results of previous executions**. In this figure, the web page listing all results is shown. For each execution, input values are reported. Results of all elaboration steps are listed and can be seen (by using a java library that must be downloaded and installed locally). A link to the diagram of the workflow is also available.

In biowep, workflow executions and related results can be saved, either temporarily or definitively, stored and later retrieved, analysed and used for further analyses. In figure 5, the web page listing all saved results and allowing for their further visualization is shown. Results can currently be displayed on the computer of the user by using a java library that must be downloaded from the portal and installed locally. A version of the java virtual machine must also be available and running on the user's side. The visualization library is derived from Taverna Workbench and it includes some extra java classes.

### Available workflows

Biowep currently includes a set of workflows that are devoted to the retrieval of data from the IARC TP53 Mutation Database [18,19] and from the CABRI catalogues of biological resources [20,21]. Some of these workflows were first created in the sphere of the Oncology over Internet – O_2_I project and have been presented in [22]. Some workflows have been made available both in Scufl and in XPDL formats. More workflows are being created and tested in various application domains.

### Support for users and developers

**Figure 6:**
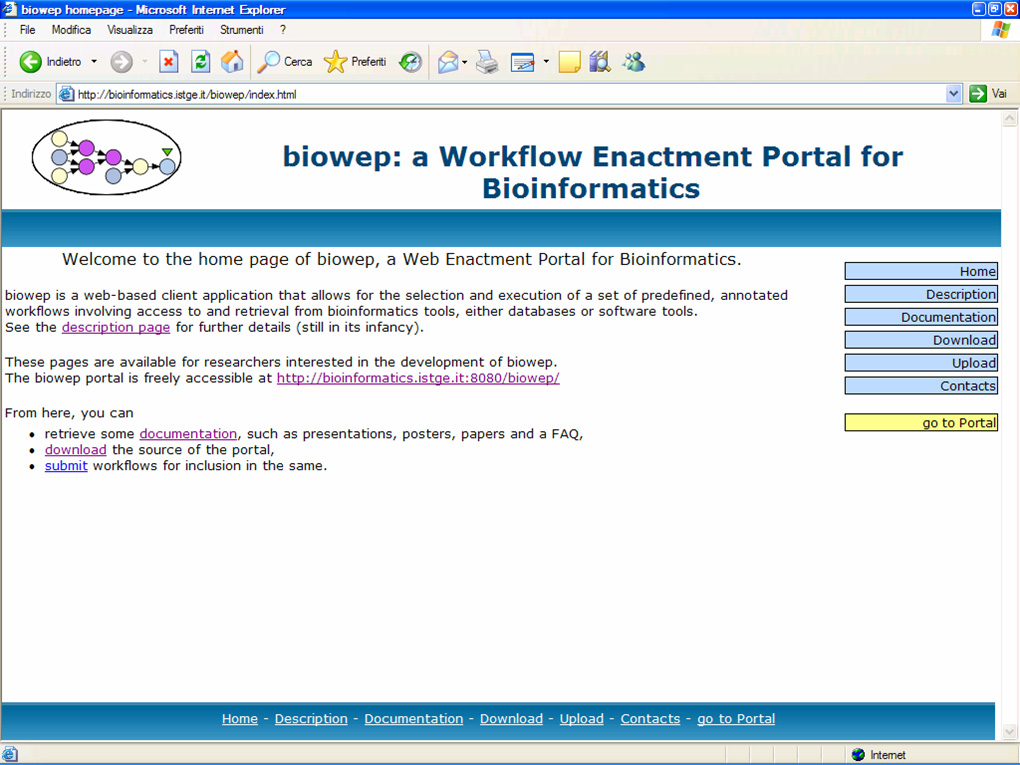
**Home page of biowep support site**. In this figure, the home page of the support web site is shown. Interested researchers can retrieve from the site all available documentation. They can also download software, database structure, workflows and submit their workflows. The same account can be used for the support site and the portal.

Support for users and developers is available in the associated site [23] from where interested researchers can retrieve all available documentation (user and installation manuals, presentations, papers) and download software, database structure and workflows (see figure 6). Archives of mailing lists are also available at the support web site. Three mailing lists have been created and will soon be announced and started: biowep-announce, biowep-forum and biowep-dev. The first is an announcements list for informing users about availability of new versions of biowep and new workflows. The second is an open discussion list on biowep features, also aimed to answer users' questions. The third list is restricted to developers and it is the depute list for discussions about improvements, new features, bug fixes.

Finally, researchers willing to submit their workflows for inclusion in the biowep repository can upload them through the *ad-hoc *form. Software download and workflows upload are limited to registered users of the portal. So, a unique registration is requested for accessing the portal and the support web site.

### Comparison with workflow engines

Biowep is not a workflow management system itself. It does not allow researchers to create their own workflows. Instead, it allows all researchers to enact predefined workflows. Biowep significantly simplifies access for not skilled researchers to automated *in-silico *procedures implemented by using external workflow management systems. This allows them to avoid undergoing a deep and continuous training on best WMS, available Web Services and their specific features and requirements. Such a training, indeed, would be needed in order to use either WMS or Web Services directly. Also, since the portal is able to enact workflows defined by different standards (currently, Scufl and XPDL) and created by different WMS (currently, Taverna and BioWMS), it offers researchers the possibility of taking profit from the best features and interoperability capacities of all included WMS.

## Conclusion

We developed a web system that supports the selection and execution of predefined workflows, thus simplifying access for all researchers. These workflows are designed to access and to retrieve data from various Web Services, that we feel are the most promising among ICT tools in view of the automation of network based data retrieval and analysis in biology. The implementation of Web Services allowing specialized software to interact with an exhaustive set of biomedical databases and analysis software and the creation of effective workflows can in fact significantly improve automation of in-silico analysis. Biowep is available for interested researchers as a reference portal. They are invited to submit their workflows to the workflow repository.

Biowep is currently being further developed in the sphere of the Laboratory of Interdisciplinary Technologies in Bioinformatics – LITBIO. Foreseen extensions of biowep include, apart from the addition of new workflows, integration with more workflow management systems and engines. We specially aim to add support for execution of workflows in a Grid network environment.

## Methods

### The system architecture

**Figure 7:**
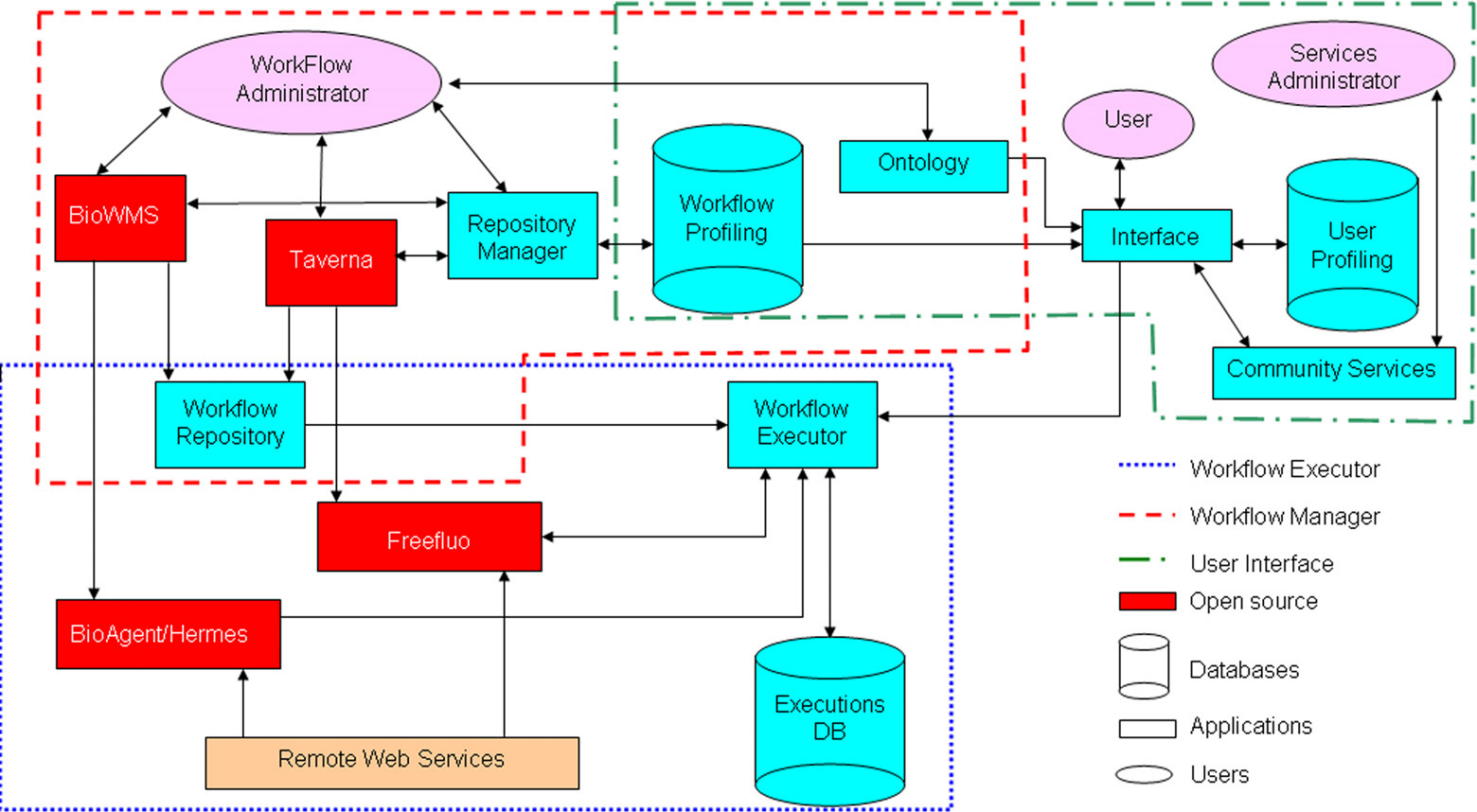
**The conceptual architecture of biowep**. The architecture of biowep includes three components. The Workflow Manager (WM) for the creation of predefined workflows by using Taverna or BioWMS, and their annotation that is done by using a classification of bioinformatics tasks (ontology). The User Interface (UI) supports end users authentication and profiling and selection and enactment of workflows. This can be carried out by searching workflows annotations. The Workflow Executor (WE) enacts workflows by using FreFluo or by submitting them to BioAgent/Hermes. In this figure, red blocks refer to open source software already available, light blue block refer to original software/database.

The conceptual architecture of biowep is shown in figure 7. It is an implementation of an extended version of the architecture designed in the Oncology over Internet project [16]. Biowep architecture includes three main components: a Workflow Manager (WM), a User Interface (UI) and a Workflow Executor (WE).

**Figure 8:**
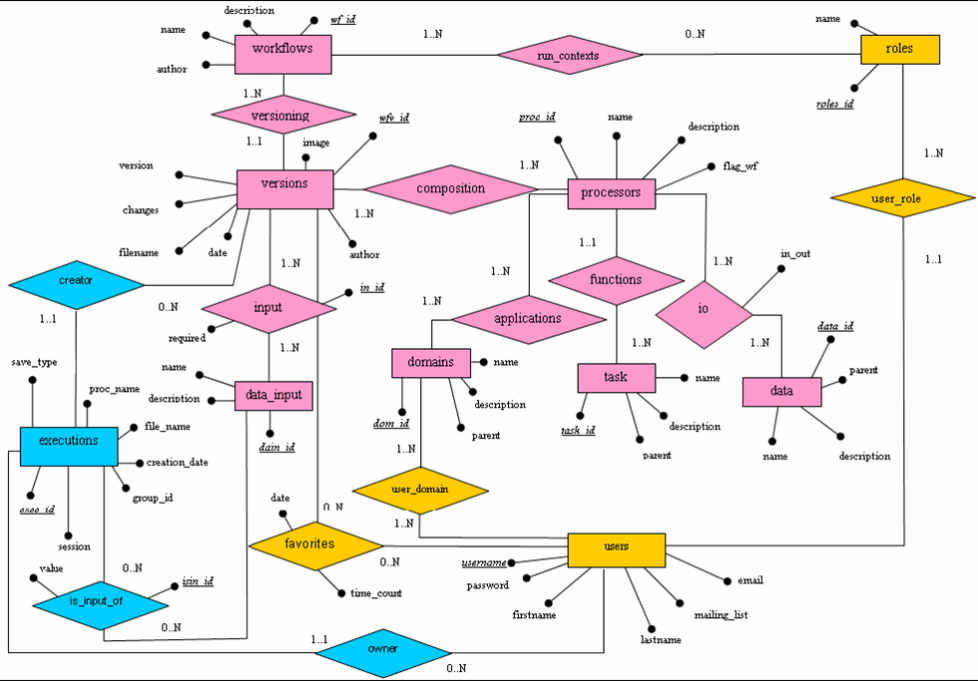
**The biowep entity – relationships – attributes schema**. In this schema, entities and relationships are coloured on the basis of their prevalent entity: yellow for data on users, magenta for information on workflows and cyan for the results of executions. It is important to note links between users' domains of interests and workflows' domains of application, as well as between users' roles and workflows' run contexts. Also, it should be noticed the difference between workflows and their versions and how the annotations and the executions data refer to versions.

The WM is external to the prototype. Its task is the creation of predefined annotated workflows. These can be created by using different WMS, on the contrary to the previous architecture that was only devoted to the Taverna Workbench [10]. Presently, the system allows for two WMS: the Taverna Workbench and the BioWMS [24]. In the former case, workflows are stored in the Simple Conceptual Unified Flow Language (Scufl), while, in the latter, the XML Process Definition Language (XPDL) format is used. We consider these formats as, respectively, the *de-facto *and *de-jure *standard. Workflows are created off-line by a Workflow Administrator and they are then entered into the system after proper testing and annotation. The role of the Workflow Administrator is also that of keeping workflows up-to-date and working by generating, when required, new versions (see database structure in figure 8 for a description of differences between workflow and version). Users which are external to the administration of the portal can also submit their preferred workflows for inclusion into the repository of the system. In this case, these are tested and annotated by the Administrator before going on-line.

The UI supports end users authentication and profiling, including the classification of users on the basis of their job/role and scientific interests. The user interface also allows for the selection and enactment of workflows. Workflows selection can be assisted by users' profiles and by searching workflows annotations. Users can request a list of all workflows in the system that have been annotated with reference to their role and/or with reference to their domains of interest.

Workflows enactment is carried out by different software. FreeFluo is used for workflows created by using the Taverna Workbench, while BioAgent/Hermes [25], a mobile agent-based middleware for the design and execution of activity-based applications in distributed environments, is used for BioWMS ones. Results of the execution of workflows can be saved and later analysed and possibly used for further analysis.

The main processing steps of each workflow are annotated on the basis of their input and output data, elaboration type and application domain. Annotations are defined by using a classification of bioinformatics data and tasks.

### The biowep database

The biowep database keeps information on users, workflows and their executions on request by users. In figure 8, the database schema is shown. In this schema, entities and relationships are coloured on the basis of their prevalent entity: yellow for data on users, magenta for information on workflows and cyan for the results of executions.

Users are registered and authenticated so that it is possible to keep trace of their executions and to store related results. The role and interest domains of the users are also archived, together with some basic data such as his/her name and email address, as a supplementary information for offering them lists of workflows that can be of their interest. Examples of the role are "computer scientist", "oncologist" or "molecular biologist", while examples of the domains of interest include "mutation analysis".

Workflows are described both on a general level (their aims and the tools involved with the elaboration) and on the implementation level (the versions of the workflow). Actual workflows, i.e. files containing all data that is needed for the execution of the workflow, are linked to single versions. Inputs that are required for carrying out the workflow are also described in details for a better support of the user entering this information. The main or most relevant steps of the workflows are annotated on the basis of a classification of tasks in bioinformatics. This classification was derived from the ontology that is available in the Taverna Workbench for describing data that is passed between processors. It has three dimensions: application domain (e.g., mutation analysis), type of elaboration (e.g., sequence alignment) and input and output data (e.g., database identifier or DNA sequence). With reference to the original Taverna ontology, main differences refer to revision of the overall structure and the addition of terms for i/o data, namely regarding biological resources and images. Our classification is still under development with the aim of transforming it in a real ontology of bioinformatics tasks.

Finally, results of executions are stored with associated data on the executed workflow and the user that requested them. Results can be stored either definitely or temporarily, in which case they are removed after a given time. All results are anyway kept at least until the end of the session during which they are generated.

### The software

Biowep is partially based on open source software, namely Taverna Workbench [26] and FreeFluo enactor engine [27]. Taverna only requirements are availability of the Java Run-time Environment [28] on either a Windows XP or Linux operating system, and, in the latter case, of the graph visualization tool Graphviz [29].

The user interface has been created by writing some java servlets and it is delivered through the Apache Tomcat engine [30].

mySQL database management system [31] is used for archiving all local data and mySQL-connector is used to get access to the mySQL database [32].

Access to the interface is therefore carried out by using any web browser. Instead, visualization of results by end users can only be carried out in his/her workstation by using a subset of the Taverna visualization library. JRE is therefore needed on the user side as well, and the needed library must be downloaded through biowep itself. To this aim, end users are requested to download the *o2i_client_lib.jar *file that includes some of the classes of Taverna Workbench (mainly visualization ones) and some original classes. This library must then be copied in a proper position in the local java implementation (usually, the jre/lib/ext sub-directory), so that the browser can find it when it is needed. More help is available on-line.

## List of abbreviations used

Biowep: Workflow Enactment Portal for Bioinformatics

CABRI: Common Access to Biological Resources and Information

DNA: DeoxiriboNucleic Acid

EBI: European Bioinformatics Institute

IARC: International Agency for Research on Cancer

ICT: Information and Communication Technologies

JRE: Java Run-time Environment

LGPL: Less General Public License

LITBIO: Laboratory of Interdisciplinary Technologies in Bioinformatics

O2I: Oncology over Internet

Scufl: Simple Conceptual Unified Flow Language

SRS: Sequence Retrieval System

UI: User Interface

WE: Workflow Executor

WfMC: Workflow Management Coalition

WM: Workflow Manager

WMS: Workflow Management System

WSDL: Web Services Description Language

XPDL: XML Process Definition Language.

## Authors' contributions

PR conceived the study, participated in its design, coordinated the development of software components and of the portal, and drafted the manuscript. GM and FDP participated in the design of the study and coordinated the development of the interface with Taverna and Freefluo. GB developed the interface with Taverna and Freefluo. EM participated in the design of the study and coordinated the development of the interface with Hermes/BioAgent. EB developed the interface with BioWMS and Hermes/BioAgent. LM participated in the design of the study. DM curated the overall installation of the components in the portal, implemented Web Services, developed workflows, and coordinated final tests. All authors read and approved the final manuscript.
